# The Inflammatory Response to Miniaturised Extracorporeal Circulation: A Review of the Literature

**DOI:** 10.1155/2009/707042

**Published:** 2010-01-13

**Authors:** Hunaid A. Vohra, Robert Whistance, Amit Modi, Sunil K. Ohri

**Affiliations:** Department of Cardiothoracic Surgery, Wessex Cardiac Institute, Southampton General Hospital, Southampton, SO16 6YD, UK

## Abstract

Conventional cardiopulmonary bypass can trigger a systemic inflammatory response syndrome similar to sepsis. Aetiological factors include surgical trauma, reperfusion injury, and, most importantly, contact of the blood with the synthetic surfaces of the heart-lung machine. Recently, a new cardiopulmonary bypass system, mini-extracorporeal circulation (MECC), has been developed and has shown promising early results in terms of reducing this inflammatory response. It has no venous reservoir, a reduced priming volume, and less blood-synthetic interface. This review focuses on the inflammatory and clinical outcomes of using MECC and compares these to conventional cardio-pulmonary bypass (CCPB). MECC has been shown to reduce postoperative cytokines levels and other markers of inflammation. In addition, MECC reduces organ damage, postoperative complications and the need for blood transfusion. MECC is a safe and viable perfusion option and in certain circumstances it is superior to CCPB.

## 1. Introduction

 Cardiopulmonary bypass (CPB) was first utilised in 1953 to repair a large atrial septal defect in an 18-year-old woman [1]. The equipment and techniques have undergone significant refinement since then and conventional cardio-pulmonary bypass (CCPB) has become the gold standard in perfusion. CCPB is generally considered safe and the mortality rates are consistently low [2, 3]. In addition, CCPB allows intracardiac procedures such as valve replacement to be performed in a blood-free field under controlled conditions. The morbidity associated with cardiac surgery is relatively high, however, with over a third of coronary artery bypass grafting (CABG) procedures reporting complications [4]. Some of these deleterious effects may be directly attributed to CCPB-induced activation of inflammation and coagulation pathways. In its most extreme form, CCPB can trigger a systemic inflammatory response syndrome (SIRS) similar to sepsis [4, 5]. This is in part related to the interface of blood components, air, and artificial surfaces within the CCPB apparatus [6, 7]. Through complex cellular and humoral interactions, SIRS following CPB can lead to coagulopathy, arrhythmias, endothelial dysfunction, neurological manifestations, and end-organ failure [5, 8, 9]. In the case of CABG, one of the methods of potentially avoiding these biosynthetic-mediated complications is to undertake beating heart revascularisation (off-pump, OPCAB). However, no significant difference between OPCAB and CCPB has been shown in terms of survival and incidence of cerebro-vascular complications. Also, technical difficulties may result in inefficacious anastomoses with a lower graft patency [10], and many “open-heart” procedures simply cannot be performed “off-pump.”

In the last decade, there have been some exciting developments in CPB design. These aim to reduce the incidence of SIRS and its complications through limiting the blood-air interface, decreasing the surface area of artificial material, and optimising the surface coating of components. Mini-extracorporeal circulation (MECC) is one example that has shown promising clinical and inflammatory outcomes. In this review, we consolidate current evidence and assess whether MECC is a viable alternative to CCPB. Below is a summary of important papers on the inflammatory response to MECC (Table 1).

## 2. The MECC System

 In a CCPB circuit, deoxygenated blood is removed from the body by venous cannulae usually inserted into the right atrium. Any blood obscuring the operating field may also be cleared with cardiotomy suction or vents. CCPB is described as “open circuit” because blood from these three sources drains into a central venous reservoir where it can freely mix with air. The blood subsequently passes through a membrane oxygenator to supply oxygen, a roller or centrifugal pump to provide an adequate arterial pressure, and an arterial filter to remove air bubbles. A heat exchanger allows the temperature of the blood infused back into the body to be controlled. In most procedures, oxygenated blood is returned to the body via the aorta.

**Figure 1:**
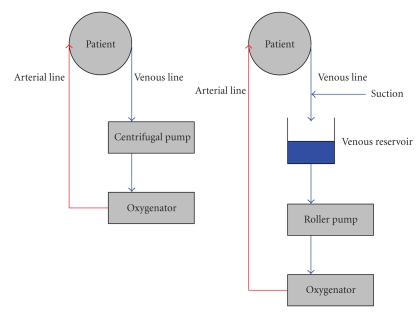
Schematic of the miniaturised extracorporeal circuit (MECC) on the left and of conventional extracorporeal circulation (CECC) on the right.

The MECC circuit incorporates a number of important differences. First, the venous reservoir has been removed making it a fully “closed” system Figure 1. As a consequence, great care must be taken to avoid air entering the venous cannulae. The lack of a venous reservoir also means that blood is not readily available to infuse, hence suction devices are not incorporated. However, a cardiotomy sucker can be incorporated into the system, if required. Consequently, blood lost into the operating field may not be directly reinfused via the system. Greater care must therefore be taken to avoid significant haemorrhage, making open-heart procedures requiring the venting of blood difficult to perform with MECC. Despite this, some circuits have been adapted to incorporate a suction device which is only activated on direct contact with liquid (optoelectrical suction). This “semi-closed” circuit has expanded the range of uses for MECC. Other centres use an independent suction device attached to a cell-saver, with postoperative autotransfusion of the scavenged erythrocytes. 

Another significant advantage of MECC is in the reduction of priming volume provided by the use of shorter lengths of tubing (480–900 ml versus 1400–1800 ml in CCPB). Less priming volume results in less haemodilution and a subsequent reduction in the need for perioperative blood transfusion. In addition, shorter tubing means that there is less surface area for the blood to interact with, which may reduce the inflammatory response. Heparinisation of patients prior to CCPB is standard, but with MECC a lower dose of heparin may be used in comparison to CCPB (150–200 IU/kg versus 300 IU/kg).

## 3. MECC and Markers of Myocardial Injury

 Damage to the myocardium during cardiac surgery is likely to be multifactorial. Ischaemia and reperfusion injury related to aortic cross-clamping as well as direct surgical trauma have been implicated in postoperative rises in cardiac-specific enzymes [22]. In addition, it has been hypothesised that the CPB machine itself contributes to myocardial injury [22]. Indeed, retransfusion of pericardial suction blood as occurs in CCPB is deemed to be an important trigger for inappropriate inflammation [23, 24]. Furthermore, damage to the myocardium may be associated with this inflammatory response [25–27]. In the largest study of its kind, Immer et al. report the results of prospective measurement of cardiac enzymes following CABG in patients undergoing CPB with either MECC or CCPB [11]. Data from some 1,257 patients were collected (MECC: *n* = 931; CCPB: *n* = 326). Patients were excluded if they had a preoperative troponin rise or a perioperative myocardial infarction. Troponin I levels, indicative of myocardial injury, were significantly lower in the MECC group at 6, 12, and 24 hours after surgery. It is worth noting that the two study groups received different cardioplegia regimens and it is conceivable that intraoperative myocardial protection was inadequate in the CCPB group. In another prospective randomised study of 60 patients, Skrabal et al. showed that patients undergoing surgery with MECC had significantly lower levels of serum troponin T and creatine kinase-MB postoperatively than those who received CCPB [22]. Importantly, the two groups in this study received the same cardioplegia regimen, which disputes the notion that inadequate cardioprotection may underlie the differences in cardiac enzyme levels. Thus, it seems that MECC may be superior to CCPB in terms of reducing the degree of myocardial injury following cardiac surgery.

## 4. MECC, Oxidative Stress, and End-Organ Dysfunction

 The reversal of periods of ischaemia can lead to reperfusion injury typified by the generation of reactive oxygen species, elevation of intracellular calcium concentrations, inflammation, and cell death [28]. CPB may result in periods of relative tissue ischaemia of the heart and of other organs, contributing to organ dysfunction and even failure [29]. Raised malondialdehyde (MDA) and an increased allantoin/urate ratio are recognised markers of oxidative stress in surgical patients [30, 31]. For this reason, Van Boven et al. measured the levels of MDA and the allantoin/urate ratios of patients undergoing surgery with both MECC and CCPB [12]. They found reduced levels of oxidative stress amongst the MECC patients following removal of the aortic cross-clamp and subsequent reperfusion. The same research group has also studied markers of alveolar damage following cardiac surgery with MECC [13]. In particular, the group looked at CC16—a Clara cell protein which may be elevated following acute alveolar injury—and showed reduced levels when MECC was used instead of CCPB. The liver is another organ prone to ischaemic injury with a reported incidence of 1.1% of severe early ischaemic liver injury following cardiac surgery [32]. This is characterised by elevated liver transaminases and carries a mortality of up to 65% [32]. Prasser et al. measured serum levels of alanine aminotransferase (ALT) and excretion of indocyanine green (a nontoxic dye metabolised solely by the liver) in 20 patients undergoing CABG and found no significant differences between MECC and CCPB groups [14]. With the aforementioned incidence of acute liver injury, it is unsurprising that this study was unable to demonstrate any difference in liver function between the two interventions. Further research into end-organ dysfunction in MECC is thus warranted.

## 5. Inflammatory Response to MECC

 CPB stimulates a systemic inflammatory response mediated through the interaction of air, blood, and synthetic components in the CPB apparatus. The inflammation is further driven by the physical trauma of surgery and the effects of ischaemia and reperfusion [6, 33, 34]. Its generation is regulated by the secretion of proinflammatory cytokines and through the activation of complement cascades [35]. Neutrophils are the predominant cell type involved in the inflammatory response after CPB, with mast cells and basophils fulfilling lesser roles [9]. Neutrophil activation can occur in response to complement or as a reaction to heparin-protamine [36]. In addition, the ischaemia/reperfusion injury causes thrombin deposition which through inflammatory cells triggers the release of cytokines such as interleukin (IL)-1, IL-6, IL-8, and the expression of adhesion molecules [9]. The ensuing SIRS can significantly derange the haemodynamic stability of patients even for long periods after the cessation of CPB, potentially increasing the time required on the intensive care unit (ICU) [37]. It is postulated that MECC could lead to a reduction in the incidence of SIRS. 

Several studies have investigated the inflammatory response triggered by MECC and compared it to CCPB. Standard postoperative measures of inflammation include leukocyte count and C-reactive protein (CRP). In a study of 400 patients, Remadi et al. reported significantly higher CRP levels in patients receiving CCPB than in those treated with MECC at 24 and 48 hours postoperatively [15]. Fromes et al. described the trend in monocyte levels intraoperatively and for a 24-hour period after [16]. They demonstrated that in both CCPB and MECC patients, the monocyte count drops following the initiation of bypass and then increases again postoperatively, peaking at 24 hours. This initial decline was attributed to the dilutional effect of commencing bypass and the later rise to the mounting inflammatory response. The drop in monocyte level was greater in the CCPB group, probably as a result of greater dilution. Interestingly, the monocyte count rose significantly less in the MECC group (*P* = .002), suggesting that a weaker inflammatory process was generated. Other authors, however, have not been able to replicate this postoperative difference in leukocyte count [17, 18]. 

Cytokines are important markers of inflammation and the levels of some are known to be raised following cardiopulmonary bypass [9, 38]. Several studies have now assessed the degree of cytokine response when an MECC system is used. Fromes et al. measured the levels of IL-1*β*, IL-6, and tumour necrosis factor *α* (TNF-*α*) at six time intervals during and after cardiopulmonary bypass (up to 24 hours postoperatively) [16]. There is a debate over whether cardiac surgery raises serum IL-1*β*, with some studies reporting a rise and others not [39–41]. Indeed, Fromes et al. were unable to detect any significant rise in IL-1*β*  levels in either MECC or CCPB patients. By contrast IL-6 levels did rise significantly, peaking 6 hours postoperatively. Furthermore, the levels of IL-6 were significantly lower in MECC patients than in those in which CCPB was used (*P* = .04). There was also a rise in serum TNF-*α*, with MECC levels again being lower than those seen with CCPB (*P* = .002). Immer et al. measured serum IL-6 and SC5b-9 at six time points in the first 24 hours following surgery in 60 patients undergoing CABG [11]. SC5b-9 is a terminal complement complex that is often raised in inflammation. The levels of IL-6 and SC5b-9 were significantly higher following surgery in the CCPB group than in the MECC group. This is further evidence that MECC is less proinflammatory than CCPB. Some authors, however, have been unable to demonstrate any significant difference in IL-6 levels in MECC and CCPB patients [17, 18]. Despite this, Ohato et al. did show significantly lower levels of IL-8 in MECC patients on day 1 after surgery [17]. 

Neutrophil elastase is a serine protease that is released during inflammation to aid in the destruction of foreign material such as bacteria [42]. The level of neutrophil elastase is known to rise after cardiopulmonary bypass, and is thought to be a marker of activated neutrophils [43]. Lower levels of neutrophil elastase infer a less pronounced inflammatory reaction. Two studies have compared the postoperative serum neutrophil elastase concentration in patients treated with CCPB and MECC [16, 17]. Both showed lower levels of neutrophil elastase in the MECC patients. This could in part be related to the reduced production of proinflammatory cytokines and the recruitment of fewer leukocytes.

## 6. Clinical Outcomes Using MECC

30-day mortality rate is a standard outcome measure in cardiothoracic surgery. To date, the literature suggests that MECC neither improves nor worsens early survival rates compared to CCPB and OPCAB [11, 16, 18–20]. Some authors have, however, reported that using MECC decreased the duration of intubation [11] and reduced the time spent on the ICU [11, 18] compared to CCPB, although some groups have been unable to replicate these findings [20]. Part of this improved early outcome with MECC could be due to a reduction in the incidence of SIRS and its complications. Atrial fibrillation (AF) is common following cardiac surgery and can occur in over 30% of patients who receive CCPB [44]. Immer et al. demonstrated an 11% incidence of postoperative AF in patients who received MECC compared to 39% in CCPB participants (*P* < .001). AF is thought to be triggered by the inflammation associated with cardiopulmonary bypass [5, 8, 9]. In a large study of the clinical outcomes of MECC, Wiesenack et al. also reported significantly lower rates of many postoperative complications, including myocardial infarction, low cardiac output, AF, pneumonia, renal failure, and cerebrovascular events [20]. Leukocyte-platelet coaggregates play an important role in ischaemia, clotting disorders, and inflammation following contact of blood with artificial surfaces [45–47]. Farneti et al. found lower levels of circulating monocyte-platelet aggregates in MECC patients [21]. They also found reduced prothrombin breakdown fragment levels and serum thrombin/antithrombin III complexes in MECC, indicative of reduced activation of the blood coagulation system. It is via these mechanisms that MECC could also result in fewer thromboembolic and bleeding complications compared to CCPB. 

The large priming volumes required in CCPB can result in significant haemodilution with low postoperative haemoglobin and haematocrit. Perioperative blood transfusions are common and are required to ensure adequate oxygen delivery to the myocardium and other organs. The haemodilution is much less pronounced when MECC is used as a consequence of the lower priming volume [11, 15, 16, 18, 20]. This can lead to a reduction in the need for blood transfusion which in itself can be proinflammatory. Indeed, Remadi et al. showed that the frequency of intraoperative blood transfusion was 6.0% in MECC patients, but was more than doubled in CCPB at 12.8% (*P* < .001) [15]. Meanwhile Fromes et al. showed that haematocrit dropped by 15.3% in CCPB patients compared to only 8.5% in MECC (*P* < .001) [11]. Haemodilution and the reduction in oxygen transportation may be a contributing factor in relative tissue ischaemia and subsequent end-organ dysfunction.

## 7. Discussion

 This review has consolidated the current literature on a new form of CPB, the mini-extracorporeal circulation system. We have paid particular attention to the role that CPB has in generating a systemic inflammatory response and have outlined ways in which MECC may be superior to CCPB. The MECC system has shown promising results with regard to cardiac damage and end-organ dysfunction. Many studies have also shown that markers of inflammation such as CRP, leucocytes, and cytokines are lower when MECC is used. Most importantly, MECC is associated with a reduction in complications, particularly arrhythmias and thromboembolic events. 

There remain some important gaps in the literature which must be addressed with future work. Many of the studies to date have large sample sizes for their clinical analyses. However, when it comes to analysing the inflammatory markers, the number of patients in each arm is often small due to the expense of undertaking these investigations. This makes it difficult to say with confidence that the patients with a deranged inflammatory response were the same ones who experienced postoperative complications. Ideally, a moderately sized randomised controlled trial would be conducted in which inflammatory markers were measured in all patients. This would enable direct correlations to be drawn between raised inflammatory markers, a SIRS response, complications, and outcome for each form of bypass. In addition, more work should look at the consequences of MECC-related inflammation and in particular markers of end-organ dysfunction, including cognitive change, renal impairment, and heart and lung injury. 

However, there are limitations of the MECC system. With CCPB, air in the venous lines can be dealt with fairly promptly. On the other hand, the same amount of air in the MECC system can lead to sudden cessation of the pump. For this reason, some surgeons apply an extra purse-string on the right atrium around the venous pipe. There is a “learning curve” associated with the use of MECC but is not a steep one and can be easily overcome. Most of the studies to date have only investigated the short-term clinical outcomes of the MECC system. If more centres are to be begin using it as standard, it would be prudent to know more about the long-term outcomes be these favourable or not. There is a paucity of randomised controlled trials highlighting long-term survival, neuro-cognitive decline, and delayed complications in this area. Despite this, MECC remains a promising alternative to conventional extracorporeal circulation especially in terms of its inflammatory results.

## Figures and Tables

**Table 1:** Summary of the important papers on the inflammatory response to MECC.

Author	Summary
Immer et al. [11]	1,053 MECC patients included.
	Reduced troponin levels in MECC.
	Reduced postoperative IL-6 and SC5b-9 in MECC.
	Lower levels of postoperative atrial fibrillation in MECC.
	Earlier extubation and shorter ICU stay with MECC.

Van Boven et al. [12]	184 participants.
	Reduced need for transfusion in MECC.
	Lower levels of MDA, allantoin/urate ratio in MECC.

Van Boven et al. [13]	20 participants.
	Lower levels of CC16 in MECC.

Prasser et al. [14]	20 participants.
	No difference in liver function tests or indocyanine green metabolism between MECC and CECC groups.

Remadi et al. [15]	400 participants.
	Higher CRP levels in the CECC group at 24 and 48 hours.
	Greater haemoglobin/haematocrit in MECC.
	Reduced need for transfusion in MECC.
	Lower postoperative troponin levels in MECC.
	Increased incidence of renal failure and haemofiltration with CECC.
	Increased incidence of focal neurological deficits with CECC.
	No difference in length of intubation/ICU stay.

Fromes et al. [16]	60 participants.
	Reduced IL-6, TNF-*α*, and neutrophil elastase in MECC.
	No difference in IL-1*β* or *β*-thromboglobulin between MECC and CECC.
	Higher levels of S100B in the CECC group.

Ohato et al. [17]	30 participants.
	Lower neutrophil elastase and IL-8 in MECC.
	No difference in white cell count, CRP, or IL-6 between MECC and CECC.
	Reduced need for blood transfusion with MECC.

Beghi et al. [18]	60 participants.
	No difference in white cell count, CRP, or IL-6 levels between MECC and CECC.

Mazzei et al. [19]	300 participants.
	No difference in IL-6, creatine kinase, and S100 between MECC and OPCAB groups.

Wiesenack et al. [20]	970 participants.
	Higher peak intraoperative lactate levels in CECC.
	Greater haemoglobin levels and lower transfusion rates in MECC.
	No difference in duration of intubation, ICU stay, or hospital stay between MECC and CECC.
	Greater incidence of myocardial infarction, stroke, atrial fibrillation, low cardiac output, renal failure, dialysis, pneumonia, reintubation, defibrillation, and rethoractomy in CECC.

Farneti et al. [21]	20 participants.
	Lower postoperative monocyte count, percentage of monocyte-platelet aggregates, and monocyte-platelet adhesion index in MECC.
	Higher prothrombin fragments and thrombin-antithrombin III complexes in CECC.
	No difference in IL-6, TNF-*α*, and *β*-thromboglobulin levels between MECC and CECC.
